# Discriminative Cut-Offs, Concurrent Criterion Validity, and Test–Retest Reliability of the Oxford Vaccine Hesitancy Scale

**DOI:** 10.3390/vaccines13121200

**Published:** 2025-11-28

**Authors:** Jonathan Kantor, Samantha Vanderslott, Michael Morrison, Robert C. Carlisle

**Affiliations:** 1 Oxford Vaccine Group, University of Oxford, Oxford OX1 2JD, UK; 2 Institute of Biomedical Engineering, Department of Engineering Science, University of Oxford, Oxford OX1 2JD, UK; 3 Center for Clinical Epidemiology and Biostatistics, Perelman School of Medicine, University of Pennsylvania, Philadelphia, PA 19104, USA; 4 Centre for Health, Law, and Emerging Technologies (HeLEX), University of Oxford, Oxford OX1 2JD, UK

**Keywords:** vaccine hesitancy, Oxford Vaccine Hesitancy Scale, cut-off, ROC, Youden’s J, Index of Union, test–retest reliability, psychometrics, COVID-19

## Abstract

Background: Validated instruments can be used to quantify vaccine hesitancy, but few provide transportable operational cut-offs or temporal stability metrics. We evaluated the Oxford Vaccine Hesitancy Scale (OVHS) to quantify discrimination for the self-reported historical COVID-19 vaccine refusal, derive and validate a single operational cut-off across populations, and assess the test–retest reliability. Methods: Five datasets (including one from a repeat administration) comprising 2451 assessments from 1989 demographically representative UK and US respondents were analyzed without pooling. Receiver operating characteristic (ROC) curves with 10,000 bootstrap replications (bias-corrected and accelerated and percentile Cis) were used to quantify discrimination. A Youden’s J near-optimal plateau algorithm constrained by a cross-dataset specificity floor (≥0.75) was used to select a transportable cut-off. A prevalence-agnostic aggregate Index of Union (Iuagg) provided secondary confirmation of this cut-off. Cut-off behaviour was visualized with a multi-sample two-graph ROC plot. The six-week test–retest reliability on a UK sample used a two-way mixed-effects, absolute-agreement ICC(A,1). Results: AUCs ranged 0.760–0.971 across datasets (derivation AUC: 0.960). The Youden plateau spanned scores 34–38; applying the specificity floor yielded an operational cut-off OVHS ≥ 35, which was confirmed by the Iuagg. At ≥35, sensitivity/specificity were 0.73–0.95/0.63–0.87 across samples; negative predictive value exceeded 0.90 in all cohorts. The test–retest reliability was good to excellent, with the OVHS total ICC(A,1) = 0.884 (95% CI 0.863–0.903); subscales ranged from 0.649 to 0.901 and the average-measure ICC(A,2) = 0.939. Conclusions: The OVHS demonstrates good-to-excellent discrimination for historical COVID-19 vaccine refusal and strong temporal stability. We found a single, transparent, and transportable operational cut-off (≥35). Our cut-off derivation framework is scale- and endpoint-agnostic and may generalize to other hesitancy instruments and decision cut-offs more broadly.

## 1. Introduction

Validated scales help investigators and clinicians better understand the contributors to vaccine hesitancy, and such scales can be used to help identify those likely to refuse vaccination, permitting tailored outreach and intervention [1,2,3,4]. Yet, while hesitancy exists on a continuum—and, indeed, that is part of the value-add of using such scales—it is also important to quantify how effectively a given scale discriminates between those who do and do not exhibit vaccine hesitant behaviours of interest, what discrete scale cut-off points are associated with historical or future behaviours, and how reliably a scale performs when provided to the same respondent at a later timepoint [5,6,7,8].

Scale discrimination has classically been demonstrated through the receiver operating characteristic (ROC) curve, and particularly by presenting the area under the curve (AUC) as a measure of discriminatory ability [9,10,11,12]. Yet clinicians and researchers may benefit from a single cut-off point that is transparently derived and is transportable across populations and time. Since most vaccine hesitancy scales present data in a continuous fashion, this leaves the cut-off choice (if any) ad hoc and vulnerable to prevalence and case-mix shifts.

Some vaccine hesitancy scales do have established cut-offs. For example, the Parent Attitudes about Childhood Vaccines (PACV) scale uses a reported cut-off of 50, which predicts under-immunization across settings [3]. Other instruments also have empirically derived cut-offs, including the 5C [6], the 7C [7], and a single-item VHA-10 [13]. Still, these cut-offs were often developed on niche populations and, importantly, their clinical utility is limited by any shortcomings of the underlying scales [2].

Similarly, most scales are validated at a single timepoint in a cross-sectional fashion, but understanding how stable the scale scores are over time—assuming there has been no change in underlying attitudes—helps support scale reliability and has important real-world implications in terms of clinical utility [14,15,16]. While select vaccine hesitancy scales—such as the Malay and Turkish versions of the PACV [17,18], the French and Urdu versions of the Vaccination Attitudes Examination (VAX) [19,20], the Polish version of the Vaccine Hesitancy Scale (VHS) [21], the Turkish version of the Oxford COVID-19 Vaccine Hesitancy Scale [22], and the Korean version of the Psychological Antecedents of Vaccination (5C) scale [23]—have been assessed for the test–retest reliability, reported intraclass correlation coefficients (ICCs) vary widely from 0.53 to 0.95 depending on the instrument, adaptation, and domain. Many of these temporal stability studies have been conducted on small, specialized populations, and indeed some have noted that the test–retest reliability is infrequently performed in vaccine hesitancy scale assessments [24].

The Oxford Vaccine Hesitancy Scale (OVHS) is a 13-item validated multidimensional instrument where scores range from 13 to 65, with higher scores suggesting higher levels of vaccine hesitancy [2]. While the initial scale development and validation work suggested its strong psychometric properties, multidimensional nature, and cross-population validity and robustness, it was initially presented as a continuous scale, while the test–retest reliability—the temporal stability of scores—was not assessed.

We therefore developed and implemented a scale-agnostic methodology to (a) assess the discrimination of the OVHS when evaluating the self-declared historical COVID-19 vaccine refusal as an outcome; (b) derive and test the performance of a single operational cut-off point for this outcome; and (c) assess the test–retest reliability of the scale. While we used the COVID-19 vaccine refusal as an example, future studies may explore whether our cut-off points remain stable when evaluating the discrimination of the OVHS in predicting vaccine refusal in other contexts and populations.

## 2. Methods

### 2.1. Samples

This study included five datasets (including one repeated assessment) on four separate demographically representative samples in the UK and US; the details of our sampling strategy have been previously reported [2,25,26,27,28]. Surveys were developed using Qualtrics (Qualtrics International, Provo, UT, USA) and we used a survey panel approach via the academic survey site Prolific Academic (Oxford, UK).

For the UK datasets, demographically representative samples were stratified by age, sex, and ethnicity (based on data from the UK Office of National Statistics). We assessed data from two distinct samples, UK_1.1_ and UK_2_; the UK_1.1_ sample was re-assessed 6 weeks after initial survey administration to assess the test–retest reliability. Our two US samples, US_1_ and US_2_, were stratified by age, sex, and ethnicity based on 2015 US national census bureau data. Since case-mix and timing differed by dataset, we did not pool data.

This study was approved by the University of Oxford Medical Sciences Interdivisional Research Ethics Committee (approval reference R81585/RE001). Study participants were reimbursed less than GBP 5/USD 5 for their time. The participants provided informed consent and were permitted to withdraw from the study at any time. Demographic details were self-declared and linked to responses using a unique 25-character alphanumeric identifier which was also used for clustering when assessing test–retest reliability. Multiple attention check items were included in the survey instrument, and responses were excluded from the analysis if a respondent failed two or more attention checks. Analyses included only the respondents with complete OVHS scores and outcomes.

Statistical analyses were performed using Stata for Mac version 16 (Stata Corporation, College Station, TX, USA).

### 2.2. Measures

The OVHS includes a total of 13 items across 3 domains, yielding a possible score range of 13–65, with higher scores reflecting greater vaccine hesitancy [2]. The theoretical neutral midpoint of this scale is thus 39, reflecting a mean item score of 3.

The reference standard for assessing the operating characteristics was defined a priori as a self-declared history of receiving no vaccinations against COVID-19, which was treated as a binary variable for unvaccinated status. Dataset-specific rates of history of COVID-19 vaccine refusal were used to calculate positive and negative predictive values (PPV and NPV).

### 2.3. ROC Curves

We generated an ROC curve for each sample and calculated the AUC [29,30,31,32]. We assessed 95% bootstrap confidence intervals for the AUC using 10,000 resamples using both bias-corrected and accelerated (Bca) and percentile methods. Resampling was stratified by outcome (refusal vs. non-refusal) and, in the UK_1.2_ sample, was clustered by participant ID to ensure within-person correlation.

### 2.4. Cut-Off Derivation Algorithms

We developed an algorithm to assess an ideal discriminatory cut-off, taking advantage of our multiple samples. Let datasets be indexed by k=1,…,K, integer cut-offs by C, sensitivity/specificity at cut-off c by $Se_{k\left(c\right)}$, $Sp_{k\left(c\right)}$, and Youden’s index J [33,34] by $J_{k}\left(c\right)=Se_{k}\left(c\right)+Sp_{k}\left(c\right)-1$. Using the UK_1.1_ dataset, *d*, as a derivation (reference), we examined all possible integer cut-offs to identify a near-optimal Youden’s J plateau around the global maximum, $J_{d}^{max}=max_{c\in C}J_{d}\left(c\right)$ with $P=c\in C:J_{d}\left(c\right)\geq J_{d}^{max}-\varepsilon$ and ε=0.01 (as we defined near-optimal as ΔJ ≤ 0.01 from the highest J). From this plateau we chose the smallest cut-off that satisfied a cross-sample specificity floor of ≥0.75 across the majority of samples—a pragmatic approach designed to prioritize sensitivity while limiting false positives, to form the admissible set $A=\{\;c\in P:\frac{1}{K}\sum_{k=1}^{K}1\{Sp_{k}\left(c\right)\geq \tau \;\}>\alpha \}$ using τ=0.75 as a specificity floor and $\alpha =\frac{\left\lceil K/2\right\rceil }{K}$ so that the floor would be met in the majority of datasets. Here, 1{⋅} denotes the indicator function (1 if the condition is true, 0 otherwise). We then selected the transportable cut-off as the smallest admissible cut-off $c^{⋆}=\min A$, which yielded OVHS ≥ 35. After fixing $c=c^{⋆}$, we evaluated the transportability in the remaining datasets by reporting operating characteristics at $c^{⋆}\;$ and, for context, each dataset’s J-maximizing cut-off; where cut-offs tied on $J_{k}\left(c\right)$ within a dataset, we report the lower c, favouring sensitivity.

A secondary ROC-based criterion was assessed using the Index of Union (IU) rule [35]; we then expanded on the published approach applicable to only a single dataset and created a novel algorithm developing an aggregate IU (IU_agg_). Briefly, for dataset k with AUC $A_{k}$ and sensitivity/specificity at cut-off c given by $Se_{k\left(c\right)}$, $Sp_{k\left(c\right)}$, we computed $IU_{k\left(c\right)}=\;\left|Se_{k\left(c\right)}-\;A_{k}\right|+\;\left|Sp_{k\left(c\right)}-\;A_{k}\right|$ selecting c that minimized $IU_{k\left(c\right)}$ with ties broken by minimizing $\left|Se_{k\left(c\right)}-\;Sp_{k\left(c\right)}\right|$. Since we sought a single transportable cut-off, we then defined a novel aggregate IU target, $IU_{agg\left(c\right)}=\;\sum_{k}w_{k}IU_{k\left(c\right)}$ with equal weights, $w_{k}$. The universal IU cut-off was the c minimizing $IU_{agg\left(c\right)}\;$ across $c\;\in \;\left\{34,\ldots ,38\right\}$.

To further visualize cut-off behaviour, we generated a multiple sample two-graph ROC (TG-ROC) style plot by plotting sensitivity and specificity against the OVHS integer cut-off for each dataset [36]. We included all available integer cut-offs from 32 to 40, connected observed points without smoothing or interpolation, colour-coded datasets, used dashed lines for sensitivity and solid lines for specificity, and marked the fixed cut-off with a vertical reference at [35].

### 2.5. Test–Retest Reliability

The test–retest reliability was performed using intraclass correlation coefficients (ICCs) with a two-way, mixed-effects, absolute-agreement single-measure model ICC(A,1). We report ICCs for the OVHS overall and each subscale in the UK population measured across two timepoints 6 weeks apart. The test–retest analysis included only participants with complete OVHS at both timepoints (*n* = 462). As a secondary analysis, we also report the average-measure ICCs(A,2), which reflect the reliability of the mean of the two OVHS administrations.

We quantified measurement precision using the standard error of measurement (SEM), computed as $SEM=SD_{T1}\sqrt{1-ICC(A,1)}$ and the smallest detectable change (SDC) beyond error, calculated as ${SDC}_{\mathrm{individual}}=1.96\sqrt{2}\times SEM$ and ${SDC}_{\mathrm{group}}={SDC}_{\mathrm{individual}}/\sqrt{n}$. ICCs were from the two-way mixed, absolute-agreement model.

## 3. Results

### 3.1. Sample Population

Our analyses incorporated data from 2451 assessments on 1989 demographically representative respondents in the UK and US. Mean ages across samples ranged from 45 to 47, and the age range of respondents was from 18 to 85. The demographic mix—age, sex, and race—of the samples reflected UK or US population-level distributions; details on these samples have been described previously [2,37]. Because samples were independent, we did not pool data for analysis. The observed COVID-19 vaccine refusal prevalence ranged from 8.4 to 20.2%.

### 3.2. ROC Curves and AUC Values

**Figure 1:**
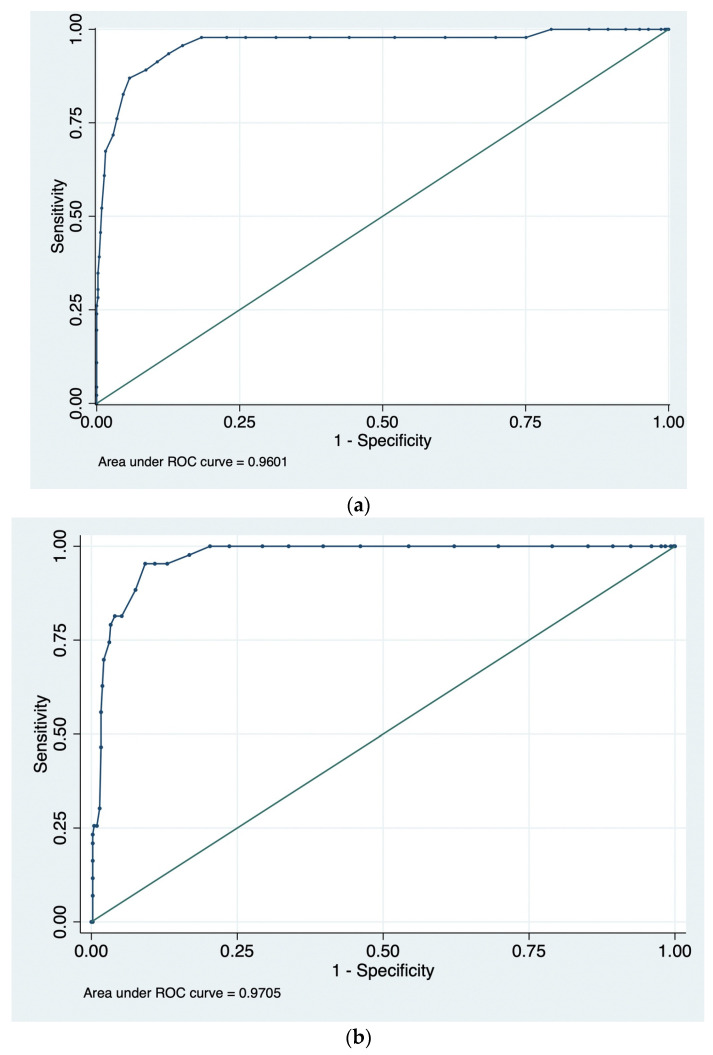
(**a**). The ROC curve for the OVHS in the UK_1.1_ dataset. (**b**). The ROC curve for the OVHS in the UK_1.2_ dataset.

The ROC curves for the UK_1.1_ (derivation) and UK_1.2_ (retest) datasets are shown in Figure 1. The AUC for the derivation sample was 0.960 (95% CI 0.890–0.981); across all samples, the AUC was the highest in the UK retest sample (UK_1.2_) and the lowest in US_2_ (Table 1). AUCs with 95% CI for the OVHS across all samples are included in Table 1 and range from 0.760 to 0.971. For reference, in our derivation sample, the AUC for the 5C short form scale [5] was 0.752.

### 3.3. Cut-Off Derivation

**Figure 2:**
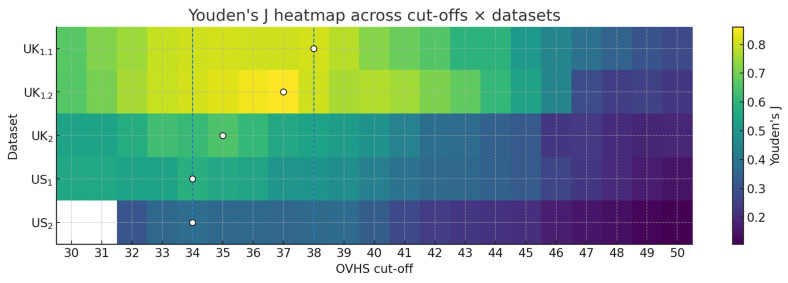
A heatmap demonstrating the near-optimal plateau. Dashed lines indicate the plateau boundaries while circles note dataset-specific J maxima. Each cell shows Youden’s J (sensitivity + specificity − 1) for a given OVHS cut-off (*x*-axis) and dataset (*y*-axis); warmer colours = better discrimination. The dashed vertical guides mark the plateau bounds (34–38) observed in the derivation set (UK1.1) where J remains within ΔJ ≤ 0.01 of its maximum (a “flat-top” optimum). Open circles mark each dataset’s J-maximizing cut-off (first occurrence, favouring the lower cut-off to preserve sensitivity). Our prespecified transport rule selects the smallest cut-off inside the plateau that meets a specificity floor (≥0.75) in a majority of datasets; this yields OVHS ≥ 35. The stable warm band from 34 to 38 indicates that small cut-off shifts change J only marginally; choosing 35 keeps sensitivity high while maintaining adequate specificity across cohorts, and in practice delivers NPV > 0.90 in all datasets, as outlined in Table 2.

In the UK_1.1_ derivation set, Youden’s J exhibited a near-optimal plateau spanning OVHS scores of 34–38, where the ΔJ was ≤0.01, with the numerical maximum at ≥38 (J = 0.812). Based on our multiple dataset selection algorithm, we chose ≥35 as the fixed cut-off as it lies on this plateau and met the predefined specificity floor criterion of ≥0.75 across the majority of datasets. A cross-dataset Youden heatmap highlighting this plateau is included in Figure 2. Youden’s J summarizes discrimination at each integer cut-off; a flat plateau means several neighbouring cut-offs perform nearly equally well. In UK1.1, J is flat from 34 to 38 (ΔJ ≤ 0.01). We then imposed a specificity floor (≥0.75) across datasets and chose the smallest cut-off in that plateau meeting the floor in a majority of cohorts, which selected ≥35. This data-driven choice is transportable and retains a high negative predictive value in every dataset (Table 2), making it suitable for screening and cross-study comparability.

As a complement to the Youden-plateau rule, we used the Index of Union to select the cut-off that keeps both sensitivity and specificity close to each dataset’s AUC—treating the AUC as the discrimination target for that cohort. Generally, $IU_{k}\left(c\right)=\left|Se_{k}\left(c\right)-A_{k}\right|+\left|Sp_{k}\left(c\right)-A_{k}\right|$ penalizes deviations of sensitivity and specificity from A_k_; lower values are better. At the dataset level, the IU minima were ≥38 (UK_1.1_), ≥37 (UK_1.2_), ≥36 (UK_2_), ≥34 (US_1_), and ≥36 (US_2_), reflecting natural cohort-to-cohort variability. When we aggregate evenly across datasets, IU_agg_ is minimized at OVHS ≥ 35 with IU_agg_(35) = 0.6626; the next-best cut-off is 36 with IU_agg_(36) = 0.6986. Thus, the IU_agg_ criterion independently confirms ≥35 and aligns with our pre-specified transportability stance (choose the smallest admissible cut-off in the near-optimal region to favour sensitivity in screening).

Youden’s J identifies where discrimination peaks but is agnostic to how sensitivity and specificity balance relative to each cohort’s overall separability (its AUC). IU_agg_ explicitly balances both margins against AUC and then sums across datasets, providing a simple, multi-cohort consensus. The small gap between IU_agg_(35) and IU_agg_(36) indicates a stable near-optimum.

Using a single cut-off of ≥35, classification performance was strong in all UK datasets and acceptable in US datasets, with NPV > 0.90 in every sample (Table 2). This supports a rule-out use case: individuals scoring < 35 are unlikely to report historical refusal, which is operationally useful for screening and triage. For completeness, Table 3 contrasts each dataset’s J-maximizing operating point with the fixed ≥35 choice and reports ΔJ; the deltas are small, consistent with the ΔJ plateau observed.

**Figure 3:**
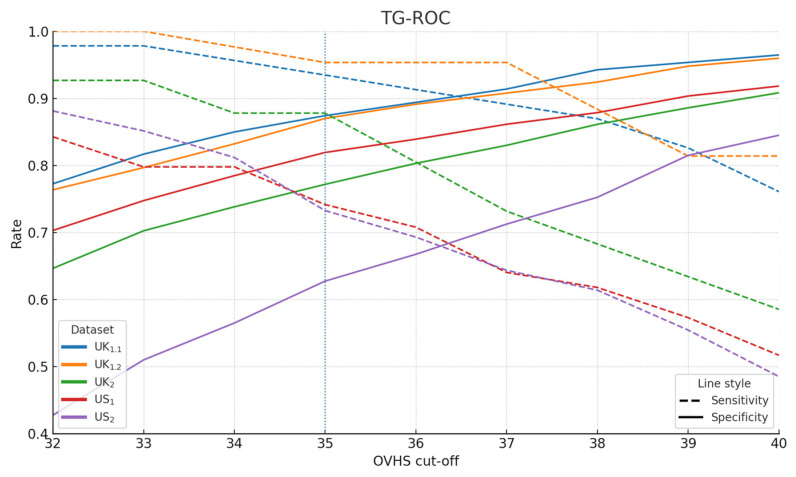
Two-graph ROC-style figure. Sensitivity (dashed) and specificity (solid) vs. OVHS cut-off across five datasets; vertical line denotes the fixed cut-off (≥35).

Figure 3 plots sensitivity (dashed) and specificity (solid) as the cut-off shifts. As expected, raising the cut-off yields lower sensitivity (fewer positives flagged) and higher specificity (fewer false positives). The cross-over neighbourhoods match the operating characteristics in Table 3. Crucially, the near-optimal band—the region where small changes to the cut-off do not materially alter discrimination—is visibly broad; the fixed cut-off ≥35 sits inside this stable range for each cohort while transporting reasonably across all five datasets. Taken together with the IU_agg_ minimum at 35, this visual highlights that ≥35 is a defensible, transparent choice for multi-setting screening.

### 3.4. Test–Retest Reliability

With a two-way mixed-effect absolute-agreement single-measure model, the overall OVHS demonstrated ICC(A,1) = 0.884 (95% CI 0.863, 0.903) in the *n* = 462 two-timepoint sample measured 6 weeks apart. The subscales showed ICC(A,1) values from 0.649 to 0.901, with lower values seen for shorter three-item subscales; the average-measure reliability was ICC(A,2) = 0.939, with the subscales ranging from 0.787 to 0.948 (Table 4).

For the OVHS total score, SEM was 2.71 points, yielding an SDC_individual_ of 7.51 points and an SDC_group_ of 0.35 points for *n* = 462 paired observations (ICC(A,1) = 0.884; SD_T1_ = 7.97). Interpreted practically, an individual’s change of ≥7.5 points over ~6 weeks likely exceeds measurement error.

## 4. Discussion

Across 2451 assessments on 1989 demographically representative respondents in the UK and US, we found that (a) the OVHS demonstrated good to excellent discrimination (ROC AUC in the derivation sample was 0.960); (b) a cross-population cut-off of ≥35 is ideal, yielding good to excellent operating characteristics across datasets; and (c) the OVHS demonstrates very good to excellent temporal stability in a UK sample with (ICC(A,1) = 0.884; ICC(A,2) = 0.939). The near-midpoint clustering of optimal operating points (34–38, with a theoretical neutral midpoint of 39) also supports the construct validity of the OVHS, as the probability of the COVID-19 vaccine refusal rises as item responses shift toward increased hesitancy, even below the neutral mean-item score. While the AUC was lower in the US samples, this reflects expected changes based on case mix and supports that the model was not overfitted. Finally, while we considered using separate cut-offs for the UK and US, the small ΔJ gains that could be seen with using two cut-offs are offset by the benefit and simplicity of a single global cut-off in these populations. Together, these data support the validity and reliability of the OVHS while also providing actionable data that could be helpful in both clinical and research contexts.

We introduce several methodological nuances that may be useful for other research groups in reporting discrimination, highlighting concurrent criterion validity, and evaluating ideal cut-off point across datasets more broadly. We include a matrix table of AUCs using computationally intensive bootstrapped confidence intervals over 10,000 resamples using both BCa and percentile methods to provide a conservative and realistic assessment of AUC values. We then used a novel Youden’s J-based algorithm to locate a near-optimal plateau of integer scores and select a cut-off by applying a small-change rule (ΔJ ≤ 0.01) within that plateau on the derivation dataset; from the admissible set we chose the smallest threshold that satisfied a cross-dataset specificity floor of ≥0.75, thereby aggregating results while also prioritizing sensitivity and high NPV for screening while limiting false positives and improving transportability. To make the selection logic transparent across cohorts, we also present a single cut-off by dataset heatmap, a compact, reproducible, and novel visual for cross-cohort comparisons. Finally, we performed a secondary, ROC-native assessment using the IU and we introduce an algorithm to derive the IU_agg_ as a prevalence-agnostic aid to cut-off derivation across multiple samples, confirming that the selected cut-off remains near the global optimum while the sensitivity and specificity are targeted toward each dataset’s AUC.

Our goal was to create a screening rule that travels reasonably across settings while remaining simple, transparent, and reproducible. A single cut-off promotes operational consistency and comparability. In the derivation dataset (UK_1.1_) we observed a near-flat Youden plateau from 34 to 38 (Δ(J) ≤ 0.01), indicating that multiple neighbouring cut-offs achieve almost indistinguishable discrimination. Our pre-specified rule therefore prioritized sensitivity/high NPV within this plateau (taking a screening stance) and added a cross-dataset specificity floor to avoid selecting a cut-off that travels poorly. The IU_agg_ then provided a multi-dataset confirmation, with IU_agg_ minimized at ≥35, aligning with the plateau-based choice.

Performance parameters varied across cohorts, as seen with the lower AUC in US_2_. Such heterogeneity is expected as prevalence and case-mix differ: PPV/NPV shift with prevalence, and sensitivity/specificity can move with changes in how the scores distribute relative to the outcome. Despite this, NPV remained >0.90 in all samples at ≥35, consistent with a rule-out orientation. We therefore present ≥35 as a default screening cut-off that preserves sensitivity and NPV across diverse cohorts, while acknowledging that false positive-averse programmes may elect to operate slightly higher (e.g., 36) at modest sensitivity cost, still within the plateau where trade-offs are incremental rather than disruptive.

Alternative approaches to creating a transportable cut-off would have included focusing on dataset-specific J_max_ value. Picking the different J_max_ values in each cohort typically yields small gains in that cohort relative to a plateau point but undermines comparability and complicates deployment. In our data, the ΔJ versus ≥35 is small (Table 3), reinforcing that plateau points are practically equivalent while the fixed rule improves consistency.

We prioritize sensitivity/high NPV in screening and therefore the cut-off of OVHS ≥ 35 flags even near-neutral scores as ‘at-risk’ to capture early or latent hesitancy that may precede refusal. A positive screen using this cut-off could therefore trigger a brief, supportive check or follow-up, while OVHS < 35 suggests a low refusal likelihood and therefore standard management in the clinical context.

We adopted a conservative approach to test–retest reliability by estimating a two-way mixed-effects, absolute-agreement, single-measure intraclass correlation—ICC(A,1) in the Shrout–Fleiss framework [38,39]—over the 6-week interval. This model treats participants as random and the two administrations as fixed, penalizing any systematic shift in means between timepoints—not only random error. This makes this approach the preferred lower-bound estimate when the goal is to judge whether a single administration at a second timepoint can be used interchangeably with the first. Confidence intervals were obtained from the F distribution, and interpretation followed standard guidance (<0.50 poor, 0.50–0.75 moderate, 0.75–0.90 good, >0.90 excellent) [40]. Because programmes may sometimes average repeated administrations (e.g., for baseline screening or research composites), we also report the average-measure ICC—ICC(A,2)—which quantifies the reliability of the mean of the two administrations; as expected from the Spearman–Brown relation [41], ICC(A,2) exceeds ICC(A,1), reflecting the precision gained by averaging. The 6-week interval was chosen to reduce recall carry-over while remaining short enough to minimize true attitude change.

This study has several other strengths. First, while many studies evaluating cut-offs and test–retest reliability focus on small samples as part of a cross-cultural validation enterprise, we include data on almost 2000 distinct respondents using demographically representative samples in the US and UK. We also evaluated performance separately across multiple independent datasets spanning countries and timepoints rather than pooling results, mirroring how programmes face case-mix and prevalence variation. Second, our ≥35 cut-off was selected from a clearly documented flat optimum and fixed before transport testing, reducing the risk of overfitting. Third, discrimination was quantified with bootstrapped CIs, operating characteristics were presented in decision-relevant terms, and the heatmap and IU_agg_ provided reproducible, cross-cohort views of cut-off behaviour. Fourth, temporal stability (the test–retest reliability) was addressed directly using absolute-agreement ICCs, documenting excellent total-score reliability and moderate-to-excellent subscale stability as well—an aspect often omitted in reliability testing.

Our work also has some important limitations. First, we used the self-reported historical COVID-19 vaccine refusal by self-report as the outcome of interest on which we assessed scale discrimination performance. Therefore, our data are subject to reporting biases and may not capture the appropriate cut-off when generalized to other vaccination campaigns. Any cut-off work is dependent on outcome definition, however, though future studies evaluating other outcomes will be beneficial. Therefore, the cut-offs outlined in this manuscript should not be generalized to predicting vaccine refusal for other vaccines until further studies are performed. Second, PPV/NPV are prevalence-dependent; while NPV remained uniformly high at our cut-off of ≥35, PPV may vary as the cut-off is applied in settings with different rates of baseline hesitancy. Third, the test–retest analysis was conducted in the UK only, and over a 6-week interval; longer time horizons and additional cross-national studies may help better characterize temporal stability. Fourth, while we provide ample evidence for the value of novel approaches to assessing validity such as our ΔJ algorithm, heatmap presentations, and the IU_agg_, these are not established approaches. We therefore present these data as secondary exploratory analyses and as confirmation of more established ROC curve, AUC, and Youden’s J-based cut-off assessments. The concordance between these approaches highlights the value of our approach and suggests it may be useful for other investigators in the future. Finally, although our survey panel sampling was broadly demographically representative and included attention checks, the population of participants who register for survey panels may differ systematically from the general population or the population of individuals seeking medical care. This limitation, however, is shared by most sampling approaches, and the large size and demographic representativeness of our samples, with generally consistent cross-dataset results, further support the robustness of our findings. Still, additional studies in different populations may be of value.

## 5. Conclusions

This study adds to the validity and reliability evidence supporting the use of the OVHS as a feasible, multidimensional scale for assessing vaccine hesitancy. Although historical COVID-19 vaccine refusal by self-report served as the worked example, our derivation framework is scale- and endpoint-agnostic so that the same steps—Youden plateau identification subject to a cross-dataset specificity floor, heatmap visualization, and confirmation using IU_agg_—can be applied to derive vaccine-agnostic or vaccine-specific cut-offs whenever a dichotomous reference is available. Indeed, these approaches could be explored more broadly when studying any algorithmic approach to cut-off derivation and have implications not only for epidemiologic research but for machine learning and artificial intelligence models where algorithmic approaches draw iteratively on diverse datasets to develop optimal decision cut-offs. Future studies addressing different outcomes of interest using cross-cultural and -language approaches may be helpful to broaden the validity evidence for the OVHS even further.

## Figures and Tables

**Table 1:** The discriminative performance (ROC AUC) of the OVHS for classifying historical COVID-19 vaccination refusal across samples.

Sample	*n*	AUC	95% CI (BCa)	95% CI (Percentile)
UK_1.1_	504	0.960	0.890–0.981	0.920–0.986
UK_1.2_	462	0.971	0.951–0.983	0.954–0.984
UK_2_	491	0.891	0.844–0.926	0.848–0.929
US_1_	500	0.851	0.799–0.891	0.803–0.894
US_2_	500	0.760	0.704–0.807	0.706–0.809

Note: AUCs are shown with 95% bias-corrected and accelerated (BCa) bootstrap CIs (primary) and 95% percentile bootstrap CIs; all CIs are calculated using 10,000 bootstrap replications with resampling stratified by outcome (unvaccinated status) and clustered by participant ID. UK_1.2_ denotes the second UK test–retest administration (~6 weeks after UK_1.1_).

**Table 2:** Discriminative performance of total OVHS score ≥ 35 for classifying self-reported historical COVID-19 vaccine refusal across five samples. Prevalence reflects the prevalence of past COVID-19 vaccine refusal (a history of never receiving a single vaccine) by self-report.

Metric	UK_1.1_	UK_1.2_	UK_2_	US_1_	US_2_
Prevalence	0.092	0.092	0.084	0.181	0.202
Sensitivity	0.935	0.954	0.878	0.742	0.733
Specificity	0.874	0.870	0.772	0.819	0.628
Youden J	0.809	0.824	0.650	0.561	0.360
Balanced accuracy	0.905	0.912	0.825	0.780	0.680
LR +	7.400	7.333	3.848	4.104	1.967
LR −	0.075	0.054	0.158	0.315	0.426
PPV	0.430	0.427	0.261	0.475	0.332
NPV	0.993	0.995	0.986	0.935	0.903

Note: UK_1.2_ includes a subset of the same subjects as in UK_1.1_ at a timepoint 6 weeks later.

**Table 3:** Operating characteristics of sample-specific optimal OVHS cut-offs based on maximizing Youden’s J for each sample.

Metric	UK_1.1_	UK_1.2_	UK_2_	US_1_	US_2_
Optimal OVHS Cut-off (≥)	38	37	35	34	34
Prevalence	0.092	0.092	0.084	0.181	0.202
Sensitivity	0.870	0.954	0.878	0.798	0.812
Specificity	0.943	0.908	0.772	0.785	0.565
Youden J	0.812	0.861	0.650	0.583	0.377
ΔJ to ≥35	0.003	0.038	0.000	0.022	0.017
Balanced accuracy	0.906	0.931	0.825	0.791	0.689
LR +	15.150	10.342	3.851	3.706	1.866
LR −	0.138	0.051	0.158	0.258	0.333
PPV	0.606	0.513	0.261	0.449	0.320
NPV	0.986	0.995	0.986	0.946	0.923

**Table 4:** Two-way mixed-effects absolute-agreement single- and average-measure ICC(A,1) and ICC(A,2) model results, with *n* = 462 over two timepoints 6 weeks apart in the UK sample. All *p*-values < 0.001.

Scale		ICC	95% CI
OVHS overall (13 items)	Single measure	0.884	0.863–0.903
	Average measure	0.939	0.926–0.949
Beliefs (7 items)	Single measure	0.901	0.882–0.916
	Average measure	0.948	0.937–0.956
Deliberation (3 items)	Single measure	0.649	0.593–0.699
	Average measure	0.787	0.745–0.823
Pain (3 items)	Single measure	0.677	0.624–0.724
	Average measure	0.807	0.768–0.840

## Data Availability

Data are available from the corresponding author.
